# Toward Development of a Label-Free Detection Technique for Microfluidic Fluorometric Peptide-Based Biosensor Systems

**DOI:** 10.3390/mi12060691

**Published:** 2021-06-13

**Authors:** Nikita Sitkov, Tatiana Zimina, Alexander Kolobov, Vladimir Karasev, Alexander Romanov, Viktor Luchinin, Dmitry Kaplun

**Affiliations:** 1 Department of Micro- and Nanoelectronics, Saint Petersburg Electrotechnical University “LETI”, 197376 Saint Petersburg, Russia; tmzimina@gmail.com (T.Z.); genetic-code@yandex.ru (V.K.); event-horizon@mail.ru (A.R.); cmid_leti@mail.ru (V.L.); 2 Institute of Highly Pure Biopreparations, 197110 Saint Petersburg, Russia; indolicidin@mail.ru; 3 Department of Automation and Control Processes, Saint Petersburg Electrotechnical University “LETI”, 197376 Saint Petersburg, Russia

**Keywords:** peptide biosensor, lab-on-a-chip, label-free detection, peptide aptamers, protein biomarkers, microfluidic biochip, troponin T

## Abstract

The problems of chronic or noncommunicable diseases (NCD) that now kill around 40 million people each year require multiparametric combinatorial diagnostics for the selection of effective treatment tactics. This could be implemented using the biosensor principle based on peptide aptamers for spatial recognition of corresponding protein markers of diseases in biological fluids. In this paper, a low-cost label-free principle of biomarker detection using a biosensor system based on fluorometric registration of the target proteins bound to peptide aptamers was investigated. The main detection principle considered includes the re-emission of the natural fluorescence of selectively bound protein markers into a longer-wavelength radiation easily detectable by common charge-coupled devices (CCD) using a specific luminophore. Implementation of this type of detection system demands the reduction of all types of stray light and background fluorescence of construction materials and aptamers. The latter was achieved by careful selection of materials and design of peptide aptamers with substituted aromatic amino acid residues and considering troponin T, troponin I, and bovine serum albumin as an example. The peptide aptamers for troponin T were designed in silico using the «Protein 3D» (SPB ETU, St. Petersburg, Russia) software. The luminophore was selected from the line of ZnS-based solid-state compounds. The test microfluidic system was arranged as a flow through a massive of four working chambers for immobilization of peptide aptamers, coupled with the optical detection system, based on thick film technology. The planar optical setup of the biosensor registration system was arranged as an excitation-emission cascade including 280 nm ultraviolet (UV) light-emitting diode (LED), polypropylene (PP) UV transparent film, proteins layer, glass filter, luminophore layer, and CCD sensor. A laboratory sample has been created.

## 1. Introduction

Noncommunicable diseases (NCDs) or chronic diseases kill around 40 million people each year, which is about 70 percent of all deaths worldwide [1]. NCDs include cardiovascular diseases, cancer, chronic respiratory diseases, diabetes, and many others. Such diseases require multiparametric combinatorial diagnostics for the selection of efficient treatment tactics [2]. This could be achieved by using biosensor systems based on peptide aptamers capable of selectively binding protein markers of diseases in biological fluids [3,4].

Currently, various instrumental methods are used in the diagnostics of NCDs. Thus, for example, in diagnostics of cardiovascular diseases, a vast range of techniques is applied: electrocardiography (ECG), echocardiography (echo-CG), computer tomography (CT), magnetic resonance imaging (MRI), stress tests (treadmill test, stress-echo), and laboratory diagnostic methods such as enzyme immunoassay, turbidimetry, etc. [5,6]. Instrumental techniques often require an accurate interpretation of the research results, which delays the diagnosis. Some are quite expensive, which limits their availability to a large number of patients [7]. Determination of molecular markers of cardiovascular diseases is an operative and informative diagnostic method, which is particularly important in treating acute conditions, enabling the early choice of a treatment strategy or surgery [7,8,9].

The biosensor according to the IUPAC, is “a device that uses specific biochemical reactions mediated by isolated enzymes, immunosystems, tissues, organelles or whole cells to detect chemical compounds usually by electrical, thermal or optical signals” [10].

Antibodies, nucleic acids, peptides, cells, etc. can be used as spatially complementary bio-recognition elements. The result of the bio-recognition process is converted into a measurable response due to various types of transducers. The most common are electronic and optical transducers. There are known examples of the implementation of biosensors using electrochemical, potentiometric, conductometric, impedimetric, voltammetric, capacitive methods, etc. [11,12]. The main feature of these methods is the modification of electrodes with various materials: graphene oxide [13], MoS_2_ nanoflowers [14], mesoporous silicon [15], gold nanostructures [16,17], zinc oxide [18], various transistor structures [19,20,21], etc. In recent years, flexible electronics technologies for manufacturing biosensors have also gained in popularity [22]. Among them, electrochemical biosensors can achieve high selectivity and sensitivity, but their performance is limited by pH, ionic strength, and temperature of the biological fluid. In contrast, optical biosensors are more universal, since they are based on detecting changes of the incidental light frequency, phase, or polarization caused by biorecognition processes, and can be divided into categories such as photometric, fluorimetric, LIF (laser induced fluorescence), colorimetric, luminescence, light scattering, surface plasmon resonance (SPR), and other types of sensor principles, which could be implemented using fiber optics, nanomaterials, diffraction grids, and other designs [23]. To ensure high sensitivity in optical biosensors, special labels are often used, for example, organic dyes, quantum dots, or nanoclusters made of noble metals [24,25]. Label-free optical biosensor systems are also known [26,27,28,29,30,31,32]. In [28], microfluidic platforms capable of the quantitative detection of biomarkers in the format of point-of-care (POC) diagnosis are described. The versatility of the microfluidics-based system is demonstrated by implementation of the chemiluminescent immunoassay for quantitative detection of C-reactive protein (CRP) and testosterone in clinical samples. The concentration range for CRP determination was 50–300 ng×mL^−1^. The authors in [29] presented design and testing of a microfluidic platform for the immunoassay. The method is based on sandwiched ELISA, whereby the primary antibody is immobilized on nitrocellulose and, subsequently, magnetic beads are used as a label to detect the analyte. It takes approximately 2 h and 15 min to complete the assay. In [30], microfluidic platforms for disease biomarker detection are described, showing recent advances in biomarker detection using cost-effective microfluidic devices for disease diagnosis, with the emphasis on infectious disease and cancer diagnosis in low-resource settings for various biomarker detection using colorimetric, fluorescence, chemiluminescence, electrochemiluminescence (ECL), and electrochemical detection. In [31], a paper-based microfluidic device was described as an alternative technology for the detection of biomarkers by using affordable and portable devices for point-of-care testing (POCT). In [32], they describe a disposable electrochemical biosensor for the determination of cancer marker CA15-3 in blood. This procedure looks too complicated for express-testing, although a high sensitivity level has been achieved. All these articles describe methods with the involvement of immunoassays, which as has been pointed out above, makes them labor intensive and demanding for reagent storage conditions. It has been also noted by some authors that when antibodies are used as biorecognition elements in biosensors, maintaining high reproducibility can be problematic due to the cross-reactivity of the detection antibody and variability in the testing medium [33].

In this paper, a label-free biosensor system for the detection of protein biomarkers in biological fluids based on fluorometric registration of the target proteins bound to the peptide aptamer was proposed and investigated. The following goals were to be achieved: (1) Selection of materials for the design of the biosensor that do not emit secondary light in response to primary UV radiation, in the range of protein fluorescence (300–350 nm); (2) The study of emission and excitation spectra of luminophores (ZnS:X) with various activators, and the selection of luminophores re-emitting the fluorescence of proteins in the longer wavelength region, which is optimal for the receiving device; (3) Development of a technology to apply a luminophore layer to an optical window; (4) Design and testing of non-fluorescent peptide aptamers with substituted aromatic amino acid residues, spatially complementary to target protein biomarkers; and (5) Draft design of the biosensor with microfluidic transport element and casing with inlet and outlet. The proposed method of biosensor signal detection will make the diagnosis of NCDs more accessible and economical as well as convenient for use in point-of-care testing (POCT) systems and personalized medicine.

## 2. Materials and Methods

### 2.1. Protein-Markers

1. Cardiac troponin I (cTpI), T9924, Sigma-Aldrich (St. Louis, MO, USA).
2. Cardiac troponin T (cTpT), T0175, Sigma-Aldrich (St. Louis, MO, USA).

The spectral selection of the detection system was carried out using bovine serum albumin (BSA) obtained from Sigma-Aldrich (St. Louis, MO, USA), CAS Number: 9048-46-8.

### 2.2. Construction Materials

The following materials were tested: polyvinyl chloride (PVC), polypropylene (PP), polymethyl methacrylate (PMMA), and cover glass.

Polyvinyl chloride (PVC), Oracle Multi Color PVC Self Adhesive Film. PVC is a thermoplastic polymer with a melting temperature of 150–220 °C. At temperatures above 110–120 °C, it tends to decompose with the release of hydrogen chloride [34]. PVC is characterized by high biocompatibility. PVC is easily combined with practically all pharmaceutical products and also endures water and chemical reactions [35].

Polypropylene (PP) film, 40 µm thick. PE is a polymer material that has good dielectric properties and low absorbance. It is not soluble in most organic solvents. PP is a transparent polymer in a wide wavelength range from UV [36].

Polymethyl methacrylate (PMMA) is a thermoplastic transparent material. Disadvantages of PMMA are a tendency to surface damage (hardness 180–190 N/mm²) and technological difficulties during thermoforming and vacuum forming, hence the appearance of internal stresses in places of bending during molding, which leads to the subsequent appearance of microcracks [37].

### 2.3. Inorganic Luminophores

The following inorganic photoluminophores were used—Green: ZnS:Cu, Blue: ZnS:Ag, and Orange: ZnS:CdS:Cu (“RPF “Luminofor” Corp.”, Stavropol, Russian Federation) [38].

### 2.4. Apparatus

Spectrofluorimetric studies were carried out using an Agilent Cary Eclipse fluorescence spectrometer (Agilent Technologies, Santa Clara, CA, USA) [39].

Protein–peptide interaction analysis was studied using an Experion™ Automated Electrophoresis Station (Bio-Rad Laboratories, Hercules, CA, USA), which performs all of the steps of gel-based protein electrophoresis, microfluidic chips, and reagents to perform protein analyses.

The biosensor reader was equipped with a camera ToupCam 5.1 MP with a color CMOS sensor Aptina MT9P006 (C) with a resolution of 2592 × 1944 pixels and 5 MP photosensitive elements. The sensor size was 5.70 × 4.28 mm. Pixel size 2.2 × 2.2 µm. Sensitivity at λ = 550 nm was 0.53 W/lux-s. Dynamic range = 66.5 dB. ADC 12 bit parallel, 8-bit RGB. S/N = 40.5 dB. Spectral range: 380–650 nm.

UV-LED: 1 W, 275 nm, SMD 4545 Deep ultraviolet LG Chip (Korea): 5–9 V, 150 mA, angle 120–140 °, radiated power 8–10 mW.

### 2.5. Peptide Aptamers

The peptide aptamers were designed using data from Protein Data Bank [40] and “Protein 3D” software, developed at the Center of Microtechnology and Diagnostics of St. Petersburg Electrotechnical University «LETI» [41,42,43]. Two versions of peptides designed for troponin T were considered. The following requirements were put forward for the peptide aptamers being developed: complementarity to the target protein, selectivity, and absence of background fluorescence. Therefore, the presented sequences had differences in the substitution of aromatic amino acids. The sequences shown were synthesized and tested by capillary electrophoresis-on-a-chip. Preparation of peptides was carried out by solid state synthesis using «Applied Biosystems 430A» instrument and in situ method with Nα-Boc-protected amino acid residues.

### 2.6. Microfluidic Chip Fabrication Process

The biosensor was designed as a sandwich structure using thick film technologies. It consisted of a substrate made of a cover glass plate (*d* × *l* × *w*: 0.2 mm × 18 mm × 18 mm), on which a polymer pattern of microfluidic channels formed. The Ordyl Alpha 350 dry film photoresist layer (*d* = 0.05 mm) was formed using standard photolithographic technology. The channels were hermitized with the polypropylene (PP) film (*d* = 0.01 mm) using aa standard lamination technique. On the top of the PP layer, a diaphragm of PLA casing (*d* = 0.4 mm) with a window for incidental UV light inlet was formatted using a 3D-printing technique and connected. The window was used to install the light filter to cut off the visible part of incidental light of the UV-LED. The other side of the glass substrate was covered with the luminophore layer for re-emission protein fluorescence in the range of visible light to which the CMOS-sensor is more sensitive. The luminophore layer was deposited using aerography technique from suspension.

The cover glass plates were treated with (3-aminopropyl)trimethoxysilane (APTMS) prior to the formation of microfluidic system topology relief in order to eliminate the nonspecific adsorption of test proteins on glass surfaces.

## 3. Results and Discussion

### 3.1. Designing Peptide Aptamers for Label-Free Detection

Peptide aptamers are combinatorial chain molecules consisting of about 15–35 amino acids, which have a high degree of affinity and specificity of attachment to the target protein molecule [44]. A design of peptide aptamers is a key problem in the development of a protein biosensor.

There are two main known groups of aptamers: oligonucleotides, most often obtained by the SELEX method [45], and peptide aptamers [46]. Since oligonucleotide aptamers do not always show sufficient selectivity for protein markers binding and demonstrate fluorescence at the UV excitation range, it seems reasonable to develop peptide aptamers, for which a number of modeling methods in silico [47,48] as well as experimental–combinatorial chemistry/high throughput analysis [49] methods are known.

The peptide aptamer search toolkit is growing and improving every year. As a resource for in vitro breeding, Ellington Lab developed the Aptamer Database, which collects catalogs of all published aptamers [49]. The database serves as a resource of information for creating aptamers for therapeutic, diagnostic, or scientific purposes.

Description of modern methods for the search and selection of peptide aptamers is presented in [50]. The described methods are mainly based on the improvement of SELEX technology and are generally very expensive and time-consuming [51].

An alternative to these methods are approaches based on computer modeling of protein structures and analysis of ligand interactions with target proteins. One of these approaches is molecular docking, which is performed by docking the ligand with the target protein binding site and moving it to determine the location and conformation that will be most beneficial for selective binding [47]. Computer simulation methods based on the principles of analysis of transient dynamic models are also used for this purpose. Based on time and length scales, these approaches can be divided into three main categories: quantum-mechanical modeling, atomic-molecular modeling, and mesoscale dynamics [52]. Monte Carlo methods and molecular dynamics are examples of atomic-molecular modeling [53]. On the other hand, the approach can be based on solving the Newtonian equation of motion in the conditions of the selected force fields, which determine the associated forces of molecular interactions. This approach was also effectively used to model biological structures and interactions between them [54]. A bead-based triple-overlay combinatorial strategy was proposed that can preserve inter-residue information during the screening process for a suitable complementary peptide to co-assemble target protein. The screening process commenced with a pentapeptide general library [55].

In this work, the peptide aptamers were designed in silico using the «Protein 3D» visualizer of supramolecular structures [41,56]. The software enables 3D structures of peptide chains to be visualized and the sites of mutual recognition to be identified. An important feature of the visualizer is the possibility of protein representation in the form of conjugated ion-hydrogen bond systems (CIHBS), considered as the basis for designing biostructures and energy transfer channels in these structures. The representation of proteins and peptides in the form of CIHBS has proved to be useful as a technology for in silico development of peptide aptamers. Based on this technology, a number of peptide sequences of 15–30 amino acid residues have been proposed and synthesized, and their ability of selective binding target proteins was experimentally tested using capillary electrophoresis [43,50].

**Figure 1:**
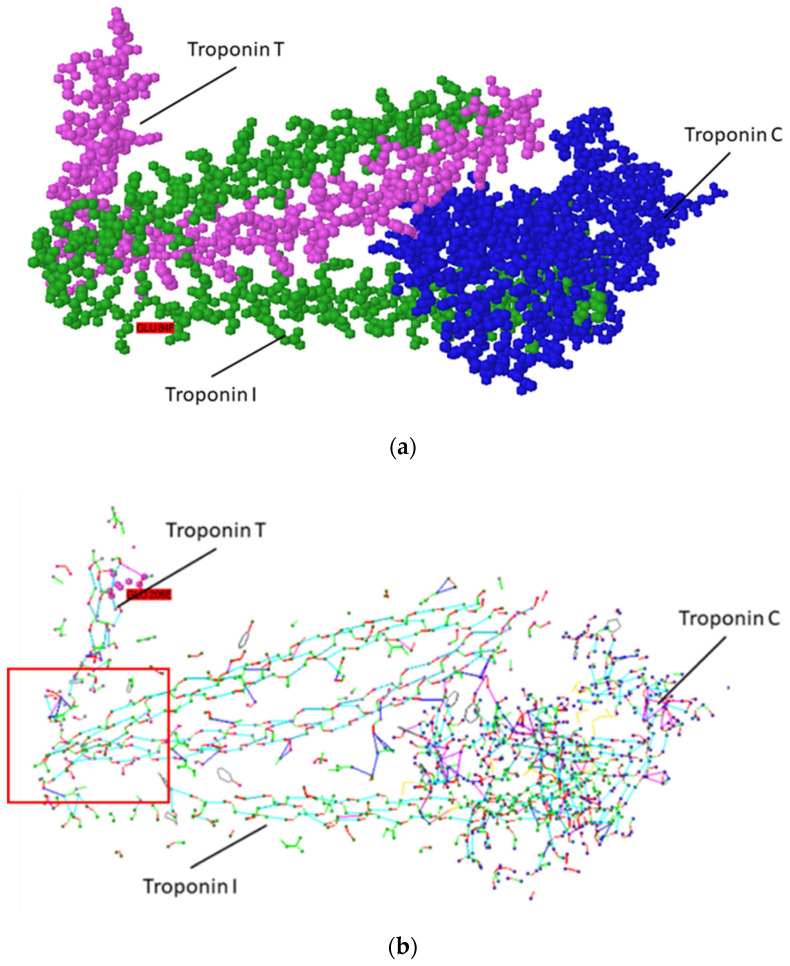
Structure of the troponin complex (**a**); troponin complex presented in the form of CIHBS rendering (**b**). Frame shows the area of contact between troponin T and troponin I, which is perspective for aptamer modeling.

To create the aptamer binding Troponin [57], a structural approach using the Protein 3D Supramolecular Structures Visualizer [44] was used to analyze the troponin complex. In addition, for the purpose of modifying the proposed sequence to replace aromatic amino acids that interfere with quantitative measurements, an attempt was made to use for this purpose the database of pentafragments developed at the CMID [42]. Protein Data Bank [40] contains files 1J1D and 1J1E of primary troponin complex structure, which is shown in Figure 1.

As can be seen in Figure 1a, troponin C is a globular molecule, and troponins T and I are extended α-helices. The troponin C molecule has multiple contacts at the ends of two other troponin subunits. However, given the multiple nature of these contacts, the use of parts of the troponin C molecule as ligands is hardly advisable. Figure 1 shows that the troponins T and I molecules have a sharp bend of the helix structure, which is located in the region of 223–226 amino acid residues. In the area of bends between individual troponin molecules, a close contact begins and continues throughout a number of amino acids, and the helix of the troponin T molecule contacts two branches of troponin I, starting from 222 atoms and ending about 260. This can be well traced when analyzing the structure in the form of CIHBS in Protein 3D software (Figure 1b; selected by frame). The region of contact of troponin subunits is very specific, since hydrogen bonds are observed between the side chains of both molecules, contributing to their mutual recognition.

From the analysis of the CIHBS structure of the troponins complex, it follows that the troponin T region from amino acids 223 to 260 is promising for the creation of an aptamer on its basis, which would allow for the isolation of troponin T. The designed and synthesized peptides LETI-2 and LETI-7 are presented in Table 1. Peptide LETI-7 is designed as a non-fluorescent twin of LETI-2 by substituting aromatic amino acid residues Trp 237, Tyr 241 and 259, Phe 248, 257, and His 223. Sequence adjustments from 224 to 258 amino acids were performed using the computer modeling, in order to reduce the number of aromatic side chains. For these purposes, pentafragments containing aromatic amino acids were introduced into the program, and the program, based on the search for pentafragments, presented amino acid variants that can substitute a given once.

Thus, the sample peptide aptamers were developed for troponin T (Figure 1) [50].

**Figure 2:**
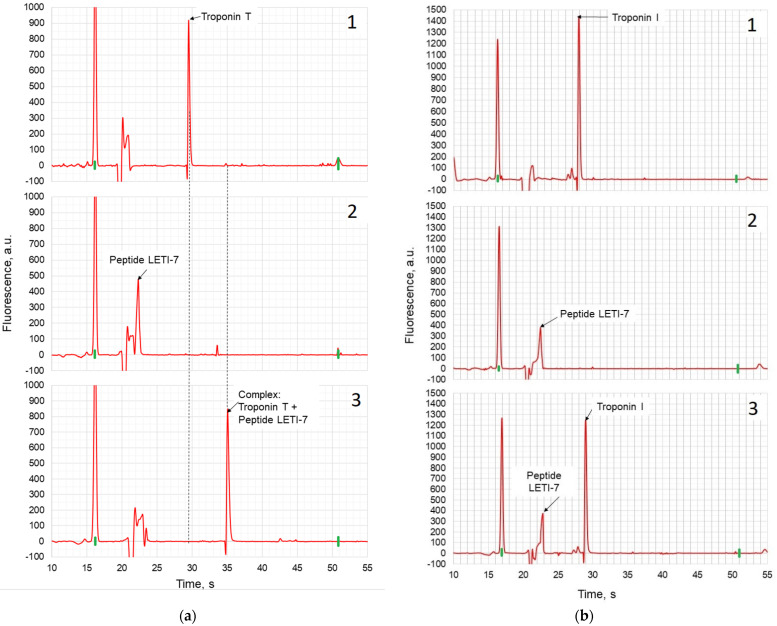
Capillary gel electrophoresis of peptide aptamer LETI-7 with troponin T (**a**) and Troponin I (**b**) using a Bio-Rad Experion station: 1—Protein, 2—Peptide LETI-7, 3—Mixture of peptide LETI-7 and protein.

The peptide aptamer with substituted aromatic amino acids (LETI-7) was tested by capillary electrophoresis-on-a-chip with a Bio-Rad Experion station. Electropherograms of troponin T, peptide LETI-7, and their mixture are presented in Figure 2a. The data show that when peptides bind to the target protein, the elution time-shifts and the area of the aptamer zone decreases. Thus, the electrophoregrams presented in Figure 2a show the formation of a complex between LETI-7 and troponin T, which manifests itself in the change of migration time of the complex in comparison with pure troponin T. The electrophoretic study of interaction between the peptide aptamer LETI-7 and the off-target protein (troponin I) demonstrated (Figure 2b) that the elution time-shifts and the area of the aptamer zone did not change, which allows us to conclude that there is no binding between the peptide and the off-target protein (troponin I).

### 3.2. Optical Scheme of Protein Detection in Biosensor Channels

**Figure 3:**
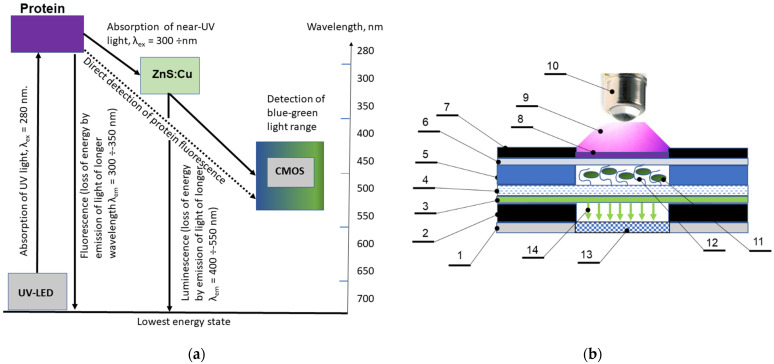
Schematic presentation of the sensing principle (**a**) and relevant optical setup (**b**) optical setup for fluorimetric analysis using a biosensor connected to a video camera. 1—casing of CMOS camera; 2—PMMA base (d = 1.5 mm); 3—luminophore layer, 4—glass filter and base of microfluidic channel (d = 0.2 mm); 5—Ordyl Alpha 350 dry film photoresist casing of biosensor with microfluidic system relief, (d = 0.05 mm); 6 –PP film, hermitization layer, and UV window (d = 40 µm); 7—PLA (d = 0.4 mm) outer casing of biosensor; 8—filter; 9—UV light; 10—UV LED; 11—protein marker; 12—aptamer; 13—CMOS-sensor; 14—generated luminescence.

In this work, we proposed to carry out direct detection of the fluorescence of biomarkers, selectively bound by peptide aptamers, without the use of special fluorescent labels (Figure 3a). The latter are labile and expensive substances and their exclusion from the protocol can increase the shelf life and ease the tests as well as reduce the cost of consumables. This is the novelty of the proposed technical solutions.

**Figure 4:**
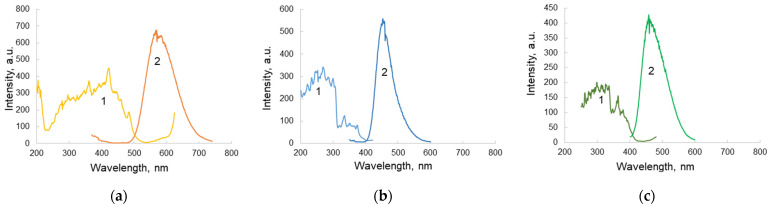
Excitation (**1**) and emission (**2**) spectra of ZnS:CdS:Cu (**a**); ZnS:Ag (**b**); and ZnS:Cu (**c**).

**Figure 5:**
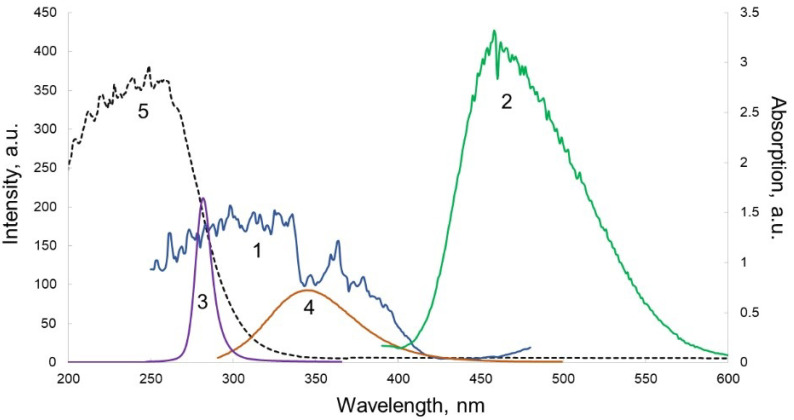
Excitation (**1**) and emission (**2**) spectra of the ZnS:Cu luminophore, UV-LED radiation spectrum (**3**) and fluorescence spectrum of BSA (**4**); (**5**) shows the range of spectrum absorbed by glass filter.

A design of the sandwich structure biosensor proposed is presented in Figure 4. The biosensor contains the luminophore layer (Figure 3b—layer 3), which is deposited on an outer surface of glass layer, thus sealing the microfluidic system of the biosensor, which is intended for the re-emission of near-UV light of protein fluorescence into the visible range of the spectrum in order to increase the sensitivity of the analytical method using a standard CMOS-sensor of the WEB camera. Three types of luminophores were tested (Figure 4) and ZnS:Cu (the green luminophore) was selected as a working component. The luminophore was deposited on the glass layer using acrylic lacquer. The spectral selection principle is presented in Figure 5 in which a coincidence of protein emission range and luminophore ZnS:Cu is demonstrated. The multilayer structure of the biosensor is presented in Figure 4, showing the topology of the 4-channel working area. The testing of the dynamic range of the biosensor was executed using BSA solution and the results are presented in Figure 5. At the present stage, the dynamic range of the sensor is about three orders.

Fluorescence of most proteins is excited at the ultraviolet range of the spectrum of λ= 220–280 nm. Proteins contain three amino acid residues that can contribute to fluorescence: tyrosine (Tyr), tryptophan (Trp), and phenylalanine (Phe). While tryptophan demonstrates the highest quantum yield of fluorescence, about 90% of all protein fluorescence is usually due to tryptophan residues. This natural fluorophore is extremely sensitive to the polarity of the environment [51]. Spectral shifts are often the result of several phenomena, among which ligand binding, protein–protein association, and denaturation can be distinguished. In addition, the emission maxima of proteins reflect the average availability of their tryptophan residues in the aqueous phase. Proteins absorb light in the vicinity of 280 nm, and the maxima of the fluorescence spectra lie in the region of λ = 320–350 nm. The decay times of the fluorescence of tryptophan residues are in the range of 1–6 ns. Tyrosine fluoresces intensively in solution, but its fluorescence is much weaker in the composition of proteins. While maximum absorption of tyrosine and tryptophan occurs at a wavelength of 280 nm, phenylalanine is excited by shorter wavelengths, and the quantum yield of phenylalanine in proteins is low, so the fluorescence of this amino acid residue is negligible in the proposed detection scheme.

Since the purpose of this work was to study the possibility of implementing a highly sensitive analysis of protein markers of acute and chronic diseases on-a-chip without the use of fluorescent labels, and on the basis of intrinsic fluorescence of protein molecules in the ultraviolet range of radiation, it is obvious that to achieve this goal, biorecognition elements (peptide aptamers) immobilized on the chip should have minimal background fluorescence. These are not applied to nucleotide aptamers, since they fluoresce when excited by blue and ultraviolet radiation. Therefore, we use peptide aptamers, especially those not containing aromatic compounds.

Thus, in this study, luminophore was used to re-emit the fluorescence of proteins in the range of 300–350 nm into the visible range of 400–500 nm. As a model, BSA solution of various concentrations was used for calibration.

Luminophores are substances that luminesce under the influence of various kinds of excitations. The method of excitation is the basis for the classification of these substances, namely: photo luminophores—excited by light. The luminescent properties of inorganic substances are associated with the formation of structural and impurity defects in the crystal lattice of the compound during the synthesis process. The former appears as a result of thermal disproportionation of the luminophore lattice and represent vacancies and ions or atoms located in the interstices of the crystal lattice. The luminescence caused by such disturbances is called non-activated (or self-activated), since its formation does not require the introduction of an activating impurity. Impurity defects appear due to the introduction at high temperatures into the crystal lattice of ions or atoms of foreign elements. The luminescence caused by such disturbances is called activated, and the activating impurities are called activators. Most of the luminophores are activated. The concept of luminescence centers is associated with activators. The chemical state of activators in the crystal lattice and the structure of luminescence centers are still the subject of numerous studies. The excitation spectra of the luminophores show the dependence of the luminescence intensity on the wavelength of the excitation light. The emission spectra of luminophores show the distribution of the luminescence energy over wavelengths. According to Stokes law, the maximum of the emission spectrum is displaced with respect to the maximum of the absorption spectrum toward longer wavelengths. Sometimes the same activator in the same substrate produces emission bands located in different regions of the spectrum. For example, the ZnS:Cu emits in the blue, green, and red regions of the spectrum, depending on the concentration of copper and the conditions for preparation. The emission spectra can depend on the intensity and wavelength of the exciting light as well as on temperature [38].

Three types of luminophores are deposited on the glass in order to achieve a band shift from 350 nm to a visible band of the spectrum in order to receive the fluorescence using ordinary CMOS sensors. In Figure 4, the excitation/emission spectra of luminophores, namely ZnS:CdS:Cu, ZnS:Ag, and ZnS:Cu, are presented.

Furthermore, based on the data of the maximum fluorescence of the protein and taking into account that the range of effective reception of the CMOS sensor covers the region of 400–650 nm, it is necessary to choose the optimal luminophore for the biochip under study.

The maximum of the fluorescence spectrum of the ZnS:CdS:Cu luminophore corresponded to a wavelength of 580 nm, which is suitable for the CMOS registration of the signal. At the same time, the excitation spectrum (Figure 4a) had a maximum at 420 nm, which is of the fluorescence range of most proteins.

The maximum of the fluorescence spectrum of the ZnS:Ag luminophore corresponded to a wavelength of 450 nm, which is suitable for the range of possible wavelengths of effective detection with a CMOS sensor, while the excitation spectrum (Figure 4b) had a maximum at a wavelength of 430 nm, which is off the range of protein fluorescence.

**Figure 6:**
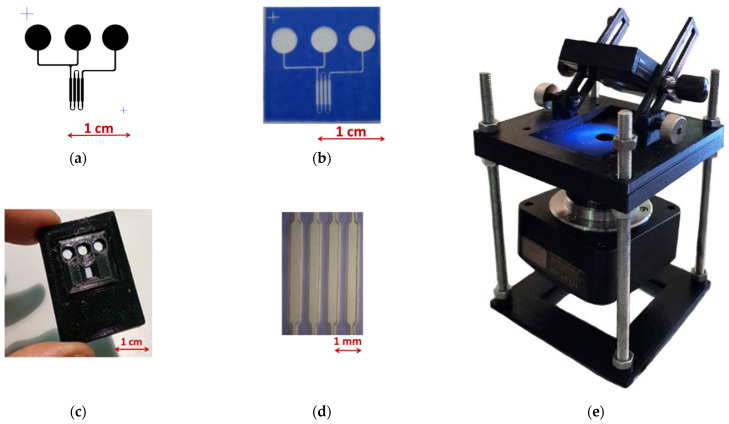
The biosensor design. Microfluidic system topology (**a**), SEM photograph of relief in the channel formed in photoresist film (**b**), view of biosensor (**c**), working channels (**d**), of the optical module for signal registration (**e**).

The green luminophore ZnS:Cu corresponded to the research objectives to the greatest extent, since its excitation spectrum had a maximum precisely at the wavelength at which we observed the maximum emission from BSA (330 nm). The fluorescence spectrum of the ZnS:Cu luminophore (Figure 4c) had a maximum at a wavelength of 480 nm and covered the range of 400–550 nm, which is optimal for most CMOS sensors in webcams (Figure 6). Quantum efficiency of this luminophore is about 90%.

### 3.3. Preparation of Biosensor Model and Its Testing

The main components of the biosensor and its assembly are presented in Figure 6. The topology of microfluidic system, comprising inlet and outlet openings for sample load, and a main channel as a negative photomask is presented in Figure 6a. The corresponding structure formed using dry film photoresist Ordyl Alpha 350 is presented in Figure 6b. Assembly of the biochip in casing is presented in Figure 6c. In the biochip, the loaded sample should move in the channel of the microfluidic system to enter the active sites of the biochip, where the «target protein–peptide» complex is formed. An inlet opening is connected to a pump. The detection area (Figure 6d) was divided into four segments intended for the immobilization of the peptide aptamers. The chip casing was formed using a 3D printing technology. The luminophore layer was applied to the glass from the suspension liquid phase. Luminophore was grated in the mill, fractionated by sedimentation, and the fraction of 1–5 µm particles was suspended in acrylic lacquer and applied to a glass substrate using the airbrushing technique. All the glass surfaces were treated with (3-aminopropyl) trimethoxysilane, which at neutral pH ensures the sufficient neutrality of the glass surface.

**Figure 7:**
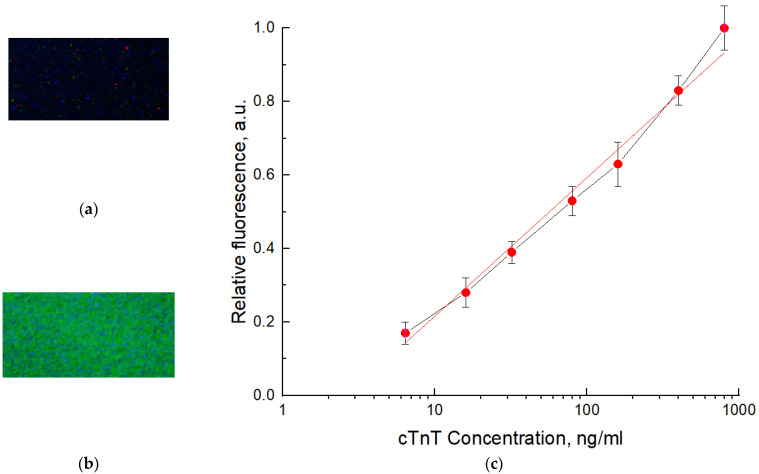
Images of the fragment of the biosensor working chamber filled with 800 ng/mL of troponin T solution recorded directly via glass window (**a**) and deposited onto the glass luminophore layer (**b**); relationship of relative fluorescence intensity versus concentration of cardiac troponin T for chips with the luminophore layer (**c**), where for curve, the red line indicates the linear approximation for the selected area.

The biosensor assembly and reader were tested with the troponin T model. Channels 50 µm deep were filled with troponin T solutions in phosphate buffer at a concentration range of 6.4 ng/mL–800 ng/mL. The images of the working area for the channel filled with troponin T solution at a concentration of 8 µg/mL for assembly with clean glass (a) and with deposited luminophore layer (b) are presented in Figure 7a,b. The concentration relationships for the relative fluorescence units (RFU) for the signal recorded for clean glass (2) and for the biosensor with the deposited luminophore layer excited with differing concentrations of troponin T are presented in Figure 7c. The relative fluorescence values were calculated as follows: RFU = (F_S_ − F_b_)/(F_Smax_ − F_b_), where F_s_ is the fluorescence signal value for the sample; F_b_ is the fluorescence signal value for the blank (buffer solution); and F_smax_ is the fluorescence of the sample with the highest measured concentration. The relationship in Figure 7c has a linear character for concentrations and satisfies the linear equation: y = 0.3769x − 0.1606, with R^2^ = 0.9886. The concentration limit of detection (LOD) for troponin T was 6.5 ng/mL, which was calculated by the standard procedure using standard deviation and slope values. The increase in sensitivity can be realized by reducing the background fluorescence, signal accumulation, and further spectral selection of the system.

Thus, Figure 7b shows the enhancement of the signal registration level due to the re-emission of fluorescence with luminophores into a longer wavelength range, where the CMOS sensor is more sensitive. The result was compared with direct detection of protein fluorescence without luminophore layer (Figure 7a) [58]. A relationship of the fluorescence intensity of the luminophore versus the concentration of protein in the working cell volume is presented in Figure 7c. The sensitivity achieved at this stage enables the quantitative detection of Troponin T, which is sufficient for the detection of most clinical levels of this biomarker in blood for diagnostics of AMI. The use of luminophore increases the sensitivity of the biosensor system by about an order of magnitude.

## 4. Conclusions

In this paper, a spectral selection strategy was studied for direct label-free detection of the protein biomarkers of diseases in biological fluids for a polymer/glass microfluidic biosensor with peptide aptamers. In recent years, the interest is growing for the development of low-cost label-free methods for biomedical diagnostics. Thus, a number of papers have been presented describing the principles of such approaches [59,60,61]. In this work, we designed, in silico, the peptide aptamer for troponin T and its non-fluorescent digital twin. The main design of a biosensor with a polymer/glass microfluidic core element was developed and tested. The layer of solid state luminophore deposited on the glass window of the biosensor enabled more than one order of magnitude sensitivity increase to be achieved in the detection of the protein procedure. The spectral selection was targeted at defining the most efficient luminophore for which the excitation range of the spectrum coincides with the emission range of the protein, and luminescence of luminophore, in turn, fits the most sensitive range of a common web-cam CMOS sensor. Finally, the ZnS:Cu luminophore was selected and deposited using acrylic lacquer. The sensitivity of the system with re-emission was tested using troponin T solution. The concentration sensitivity of protein detection achieved at this stage was 6.5 ng/mL with the optical path length of 50 µm.

Thus, in accordance with the tasks set, based on the structural and technological features of the optical planar element, the following studies were completed:

1. The fluorescence of a number of polymer materials was studied under excitation in the range of 260–280 nm, in order to select materials with minimal background fluorescence under radiation. The results showed that the selected materials meet the necessary requirements.
2. A material for the preparation of the inlet window of the biosensor, transparent for UV radiation at 275 nm, was selected. The most fitting proved to be a PP film, 100 µm thick.
3. A peptide aptamer constructed using the «Protein 3D» software complementary to troponin T was designed. On its basis, its non-fluorescent digital twin was designed, which retained its three-dimensional complementarity to target protein, which has been shown with capillary electrophoresis-on-a-chip experiments.
4. For the outlet window transmitting fluorescence of troponin T for the channel depth of 50 µm in the range of 300–350 nm and absorbing incidental UV radiation at 275 nm, the most fitting material was glass. The cover glass plates were used for the preparation of this element.
5. Comparison of solid-state inorganic luminophores in order to select the optimal material for use in a planar optical element with an excitation range of 300–350 nm (Troponin T emission range) and an emission range of 450–550 nm (optimal for a receiving device) showed that a luminophore with a ZnS:Cu composition was the most consistent with the tasks.
6. A laboratory sample of the biosensor was designed and manufactured. Technology for applying a luminophore to glass was developed.

Further directions of the low-cost label-free system development are as follows: the development of improved miniature optical arrangements enabling a decrease in background light from the luminophore; improvement of software for signal capture and processing, and enabling signal accumulation during the capture process, etc.

## Figures and Tables

**Table 1:** List of synthesized peptides.

Name of Peptide	Structure	Number of Amino Acid Residues
LETI-2	HLNEDQLREKAKELWQTIYNLEAEKFDLQEKFKQQKE	38
LETI-7	TLNEDQLREKAKELAQTIANLEAEKIDLQEKAKQQKYE	
